# Relationship Between Scarring and Dog Aggression in Pit Bull-Type Dogs Involved in Organized Dogfighting

**DOI:** 10.3390/ani6110072

**Published:** 2016-11-15

**Authors:** Katherine A. Miller, Rachel Touroo, C. Victor Spain, Kelly Jones, Pamela Reid, Randall Lockwood

**Affiliations:** 1 Anti-Cruelty Behavior Team, American Society for the Prevention of Cruelty to Animals, New York, NY 10128, USA; pam.reid@aspca.org; 2 Forensic Sciences Department, American Society for the Prevention of Cruelty to Animals, New York, NY 10128, USA; rachel.touroo@aspca.org (R.T.); randall.lockwood@aspca.org (R.L.); 3 Shelter Research and Development Department, American Society for the Prevention of Cruelty to Animals, New York, NY 10128, USA; vic.spain@aspca.org; 4 Cummings School of Veterinary Medicine, Tufts University, North Grafton, MA 01536, USA; kelly.jones@tufts.edu

**Keywords:** dogfighting, dog, aggression, veterinary, behavior, forensic, scar, wound, animal cruelty

## Abstract

**Simple Summary:**

Organizations responsible for placing dogs seized from dogfighting investigations often must determine if a particular dog should be euthanized because it is too dangerous or if it is safe to place the dog in an adoptive home. In this study, we examine whether the extent of scarring from dog fighting is a reliable predictor of aggression towards other dogs and therefore could be used to help make that decision. We found that dogs with 10 or more scars in the three body zones where dogfighting injuries tend to be concentrated were more likely, on average, to show aggression to other dogs. The relationship is imperfect, however. Many unscarred dogs were dog aggressive while some highly scarred dogs were not. Therefore, we recommend also assessing a dog’s behavior before making decisions about its disposition.

**Abstract:**

When pit bull-type dogs are seized in an investigation of organized dogfighting, heavily scarred dogs are often assumed to be highly dog aggressive due to a history of fighting. These dogs may be deemed dangerous and euthanized based on scarring alone. We analyzed our existing data on dogs seized from four dogfighting investigations, examining the relationship between the dogs’ scars with aggression towards other dogs. Scar and wound data were tallied in three body zones where dogfighting injuries tend to be concentrated. Dog aggression was assessed using a model dog and a friendly stimulus dog in a standardized behavior evaluation. Scarring and dog aggression were significantly related, more strongly among male (Fisher’s Exact *p* < 0.001) than female dogs (Fisher’s Exact *p* = 0.05). Ten or more scars in the three body zones was a reasonable threshold with which to classify a dog as high risk for dog aggression: 82% of males and 60% of females with such scarring displayed dog aggression. However, because many unscarred dogs were dog aggressive while some highly scarred dogs were not, we recommend collecting behavioral information to supplement scar counts when making disposition decisions about dogs seized in dogfighting investigations.

## 1. Introduction

Organized dogfighting is rampant in many parts of the United States even though it is a felony offense in all 50 States. The American Pit Bull Terrier is the breed most commonly associated with organized dogfighting in this country [1,2,3].

Dogs that have been pitted against other dogs typically display scars due to the wounds caused by the teeth of their opponent. The scarring associated with organized dog fights is primarily concentrated on the front legs, head, and muzzle. This is a different pattern of injury than that sustained in spontaneous, non-organized fights between dogs, as documented in an earlier study [4] and supported by the current study. This is because dogs in organized fights face each other while fighting. In fact, the instruction given by the referee in such fights is “face your dog,” initiating the mutual attack. In addition, while fighting dogs are selected and trained to cause injury to their opponent, non-organized fighting amongst dogs is more likely to consist of ritualized displays and non-injurious biting [5]. Any injuries that do occur in a spontaneous fights among dogs of the same sex and of similar size are primarily located on the pinnae (ears), dorsal and lateral neck, and front legs—although to a lesser extent than in an organized fight [4].

In our experience, heavily scarred pit bull-type dogs are often assumed to be highly dog aggressive as the result of a history of fighting. However, we have found that not all dogs seized from dogfighting investigations, including some bearing scars, are aggressive to other dogs. This may be because the dog is in the early stages of its training as a fighting dog and may still retain non-aggressive, social behavior toward other dogs. On the other hand, we have identified some dogs with no or few scars that exhibit significant dog-aggressive tendencies. These dogs are likely young, inexperienced dogs who have not yet been fought, or are dogs that have only been “rolled” (a training fight) once or twice, or breeding dogs who were not used for fighting but possess a strong genetic propensity for aggressive behavior.

It is our experience that some organizations responsible for placing dogs seized from dogfighting investigations do not have the resources to conduct thorough behavior evaluations on the dogs and therefore may rely on extent of scarring to form their decisions on the disposition of the dogs. Highly scarred dogs are usually assumed to be dog-aggressive, although there is currently no commonly accepted threshold for the number of scars on which to base this determination. Other organizations who do conduct behavior evaluations may utilize the extent of scarring as a secondary piece of information, to supplement their disposition decisions, especially with respect to dogs that do not show aggression toward other dogs but also do not engage in friendly behavior. Having information concerning the validity of utilizing the extent of scarring as a proxy for dog aggression would assist these organizations in making more informed decisions, and potentially reduce the euthanasia of non-dog aggressive pit bull-type dogs.

To our knowledge, no analysis has been conducted to determine the relationship between the presence and extent of wounds or scarring and dog aggression among dogs seized from dogfighters. We analyzed this relationship with the following objectives:

1. Describe the extent and distribution of scarring among dogs seized from organized dogfighting investigations;
2. Describe the extent of aggression towards other dogs exhibited by dogs in this population;
3. Analyze the relationship between extent of scarring and aggression towards dogs;
4. Investigate whether the sex of the dog affects the relationship between scarring and dog aggression;
5. Determine whether level of scarring is a useful screening test/proxy for aggression towards dogs and, if so, what threshold of scarring is appropriate for identifying dogs at high risk for dog aggression?

## 2. Materials and Methods

The American Society for the Prevention of Cruelty to Animals (ASPCA) Anti-Cruelty Behavior Team and Forensics Sciences departments deploy nationally to large scale cases of animal cruelty, including dogfighting. Data were collected as part of our routine documentation of dogfighting evidence. We collected data from 279 pit bull-type dogs seized as evidence in four organized dogfighting investigations conducted from 2012 through 2015. The dogs originated from 13 different owners residing in six States in the Southeast and Midwest United States.

In order to be included in the analyses, dogs must have had complete forensic behavioral and medical data available. Dogs included in the study were estimated by veterinary examination to be young adults (six months to three years) or adults (at least three years), based on eruption of all permanent teeth and the degree of dental wear. Puppies less than six months old were excluded from analyses because such dogs are not used in organized fights and do not display scarring consistent with organized dogfighting activities.

Behavioral data were collected during the course of standardized evaluations conducted by teams of forensic animal behavior professionals who were Certified Professional Dog Trainers (CPDT) or Certified Applied Animal Behaviorists (CAAB). These evaluations took place at ASPCA temporary shelters where the dogs were housed and cared for until disposition was granted to the ASPCA by the courts. After they were transferred from their point of origin, the dogs were given at least three days to settle into their new environments before their behavior was assessed. Dogs who were ill, debilitated, or in late pregnancy were not behaviorally evaluated until their physical condition stabilized, as determined by the ASPCA’s veterinary medical team.

In the evaluation, dogs were exposed to a variety of situations that simulated what they would experience in a typical shelter or home environment, including being petted and handled, having food and a rawhide chew taken away, being scolded, meeting a toddler-sized doll, and greeting a friendly dog.

**Figure A1:**
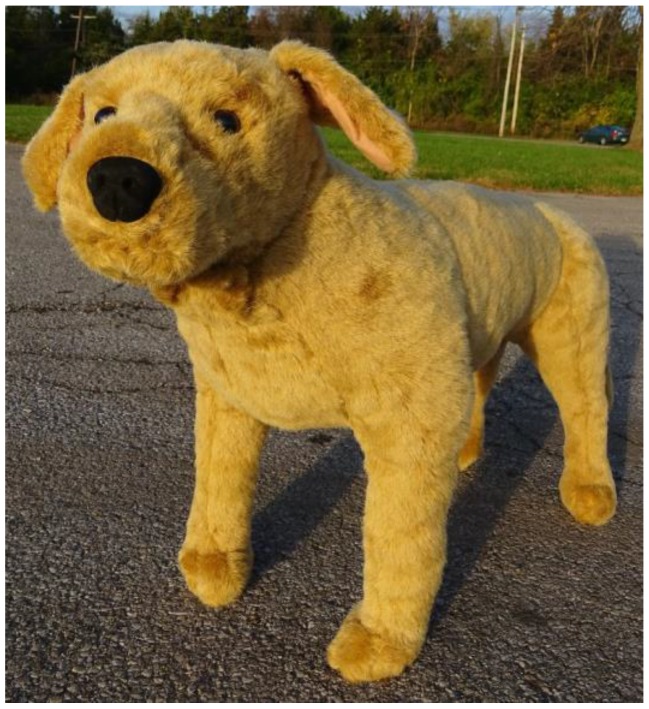
Photograph of life-size model of a Labrador retriever used for the Model Dog Test.

A previous study demonstrated that, for 81% of dogs from dogfighting cases, an aggressive reaction to a life-size model of a Labrador retriever dog matches their reaction to a real dog [6]. (See Figure A1 in Appendix A for a photograph of the model dog). For safety reasons, a similar model dog was used in our behavior assessments to determine whether to introduce the test dog to a friendly stimulus dog. A display of classic dogfighting behavior to the model dog (see Table 1) was sufficient to halt further testing, precluding an introduction to the stimulus dog. If the test dog exhibited any other reaction to the model dog however, we proceeded to introduce it to the stimulus dog.

The stimulus dog was chosen for its tendency for social, non-aggressive behavior towards other dogs. For logistical reasons, and to increase the generalizability of results, multiple stimulus dogs were used throughout this study, but each test dog met only one stimulus dog. The stimulus dog was always the same sex as the test dog. The stimulus dog was first paused outside the gate of the evaluation area so the dogs could meet initially through a fence. The stimulus dog was then brought into the pen, with both dogs on leash, to assess the test dog’s reaction from a distance for a few seconds. Lastly, the test dog was permitted to meet the stimulus dog nose-to-nose on leash, wearing a muzzle if caution dictated. Aggression on the part of the test dog at any point was sufficient to halt further testing. If no aggression was observed, the two dogs were permitted to interact on leash for 1–3 min. Behaviors recorded during the model and stimulus dog tests utilized in this study are defined in Table 1. When aggressive as well as non-aggressive behavior was displayed, such as arousal followed by offensive aggression to the stimulus dog, we categorized the interaction according to the aggressive behavior.

**Figure 1:**
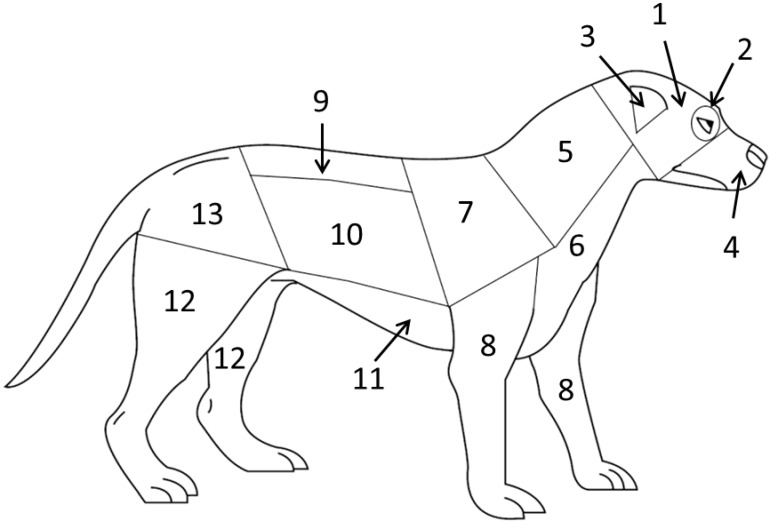
Body surface zones used for categorizing location of wounds and scars [4]. The 13 body surface zones are: (**1**) the dorsal and lateral head; (**2**) the eye and periorbital region; (**3**) the pinnae; (**4**) the muzzle and oral mucosa; (**5**) the dorsal and lateral neck; (**6**) the ventral neck and chest; (**7**) the scapular region; (**8**) the front legs; (**9**) the thoracic and lumbar spine; (**10**) the lateral thorax and abdomen; (**11**) the ventral thorax and abdomen; (**12**) the hind legs; and (**13**) the pelvis and tail. Figure used with permission.

**Figure B1:**
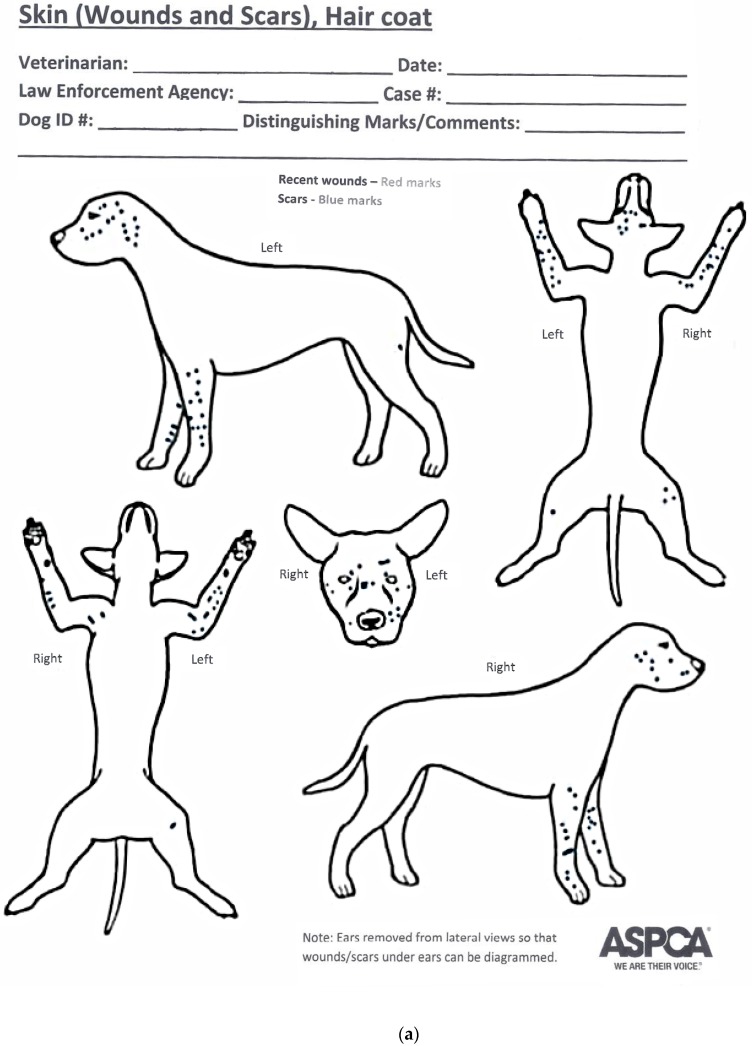

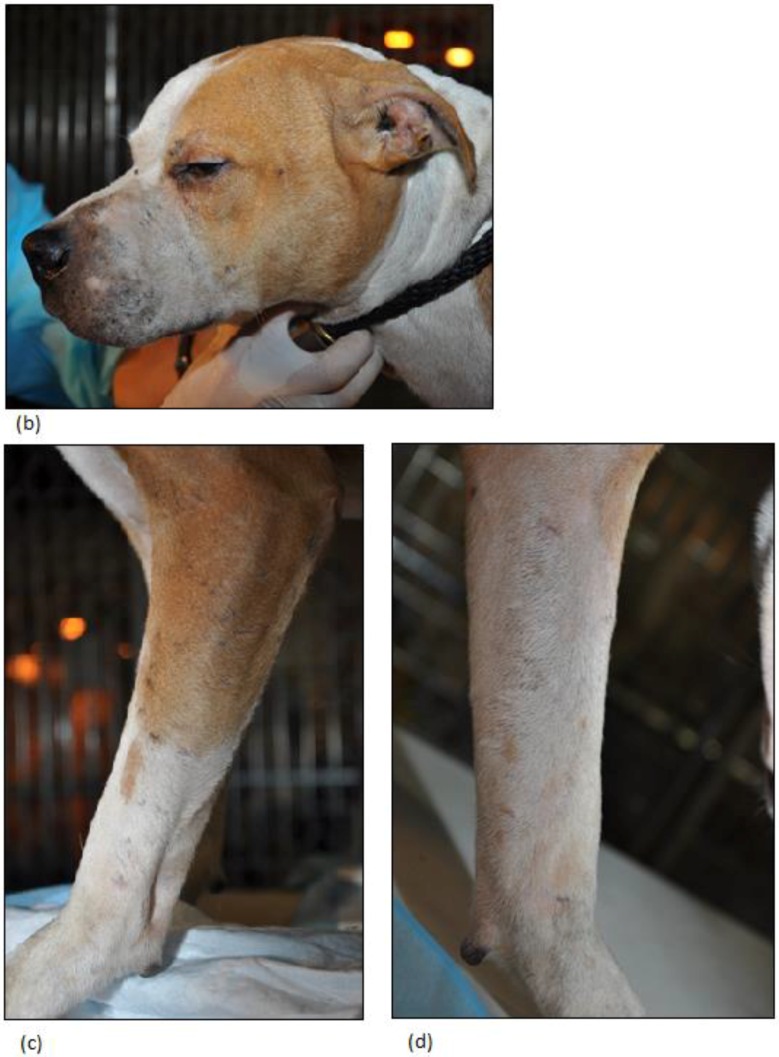
Sample scar chart and photographs of the wounds depicted. (**a**) Scar chart; (**b**) Scarring on left lateral aspect of head; (**c**) Scarring on lateral (outside) aspect of the left front leg; (**d**) Scarring on the medial (inside) aspect of the left front leg. A template for the scar chart is provided as Figure S1.

Medical data were collected during the course of standardized forensic medical exams conducted by veterinarians in the ASPCA’s Forensic Sciences department. Exams were initiated upon the dogs’ arrival at the ASPCA’s temporary shelters. We categorized the extent of each dog’s injuries by location in 13 different zones on the body (see Figure 1) using a standard data collection sheet, commonly called a “scar chart” (see Figure B1 in Appendix B). Such categorization was based on results from a previous study, which established that the distribution and extent of scarring on dogs involved in organized dogfighting are significantly different than that of dogs involved in naturally occurring fights [4]. Dogs involved in organized dogfighting sustain a higher prevalence of injuries on the front legs, dorsal and lateral head, and the muzzle and oral mucosa. Therefore, we summed the number of injuries, including both scars and recent wounds, in these three body zones as well as the total number present on the body. All visible indications of wounds and scars were counted, including older scars that were sufficiently healed such that they appeared as an area of alopecia or depigmented hair coat (white hair regrowth).

The primary behavioral outcome for analysis was dog-directed aggression, defined by demonstrating classic dogfighting behavior to the model dog or offensive aggression to the stimulus dog. For each dog, the total number of scars (including recent wounds) in the three body zones was categorized as: no scars, 1–9 scars, 10–39 scars, or ≥40 scars. These thresholds were selected to ensure that each category had a sufficient number of dogs for meaningful statistical analyses and also for ease of interpretation. The scar-count category was treated as a nominal variable (not ordinal) for analytic purposes. This choice is more conservative statistically and was appropriate in the event of subgroups for which there was not a consistent trend in the relationship between scarring category and dog aggression.

We calculated exact 95% confidence intervals for the proportion of dogs with each behavioral response using the Clopper-Pearson method [7]. In order to assess whether the relationship between scarring and behavior may differ by sex, the proportion of dogs with each behavioral outcome was also stratified by sex. The overall association between level of scarring and behavior was measured using Fisher’s exact test.

Receiver operator characteristic (ROC) curves [8] were created to determine which thresholds of scar count (summed from the three selected body zones) are most useful as a screening test for classifying dogs as high risk for dog aggression, in the absence of a full behavioral assessment. The area under the curve (AUC) was used as a measure of overall accuracy of using scar data, and it was calculated separately for male and female dogs. For the threshold that appeared to offer discrimination, we calculated sensitivity, specificity, positive predictive value (PPV), and negative predictive value (NPV) stratified by sex. Where relevant, 95% confidence intervals were calculated using the Clopper–Pearson method. All analyses were conducted in Stata 13.1 (StataCorp LP, College Station, TX, USA).

## 3. Results

Of the 279 dogs from which data were collected, 24 were excluded from analysis because they did not receive a complete behavioral assessment and 3 were excluded because they were under 6 months old. Among the remaining 252 dogs that qualified for analysis, 54% were female and the majority (60%) were estimated to be adults at least three years of age (Table 2). 180 dogs (71%) had one or more scars. Among these, the median number of scars (including wounds) was 36.5 (range: 1 to 256). The remaining 29% of dogs had no scars at all. Overall, 14% of dogs had a total scar count between 1 and 9, 23% between 10 and 39, and 34% had 40 or more scars (Table 3). The majority of dogs had one or more scars in the three zones commonly scarred during dogfighting: front legs (63%), dorsal and lateral head (57%), and muzzle and oral mucosa (51%). Only 26 dogs (10%) had both healed scars and recent wounds in these three body zones, and no dogs had only recent wounds without any scars. All further results rely on the sum of scar data, including both scars and recent wounds, tallied from these three body zones.

**Figure 2:**
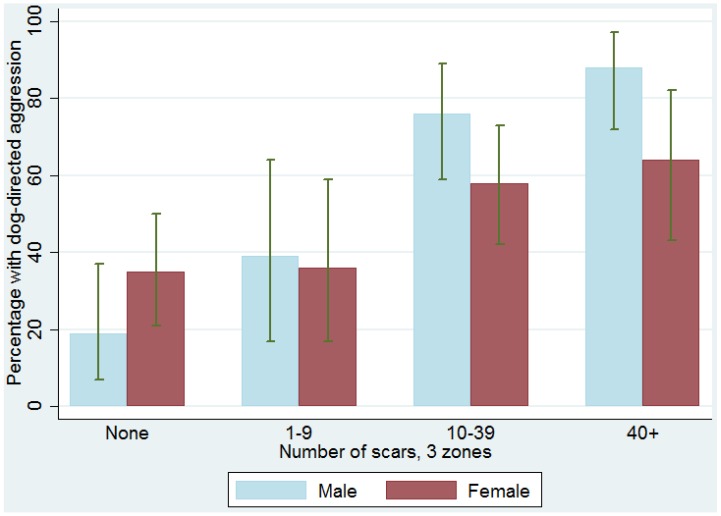
Percentage of dogs displaying dog aggression, by scar count and sex, with 95% confidence intervals.

We found a linear relationship between the level of scarring and frequency of dog-directed aggression, with a stronger relationship among males (Fisher’s Exact *p* < 0.001) than females (Fisher’s Exact *p* = 0.05). Among males, the proportion showing dog-directed aggression ranged from 19% among those with no scars to 88% among those with ≥40 scars. (Table 4 and Figure 2). Among females, the proportion showing dog-directed aggression ranged from 35% among those with no scars to 64% among those with ≥40 scars. The presence of both wounds and healed scars on a dog may be considered an indication of participation in multiple dog fighting episodes. When classifying recent wounds separately from healed scars in these body zones, we found the proportion showing dog-directed aggression ranged from 29% among those dogs with neither wounds nor scars, to 62% for those with only healed scars, to 69% for those showing both wounds and healed scars. Because analyzing scar and wound data separately did not provide better prediction of dog aggression than using the sum of both wounds and healed scars, we chose to use the sum of both wounds and healed scars in all analyses.

**Figure C1:**
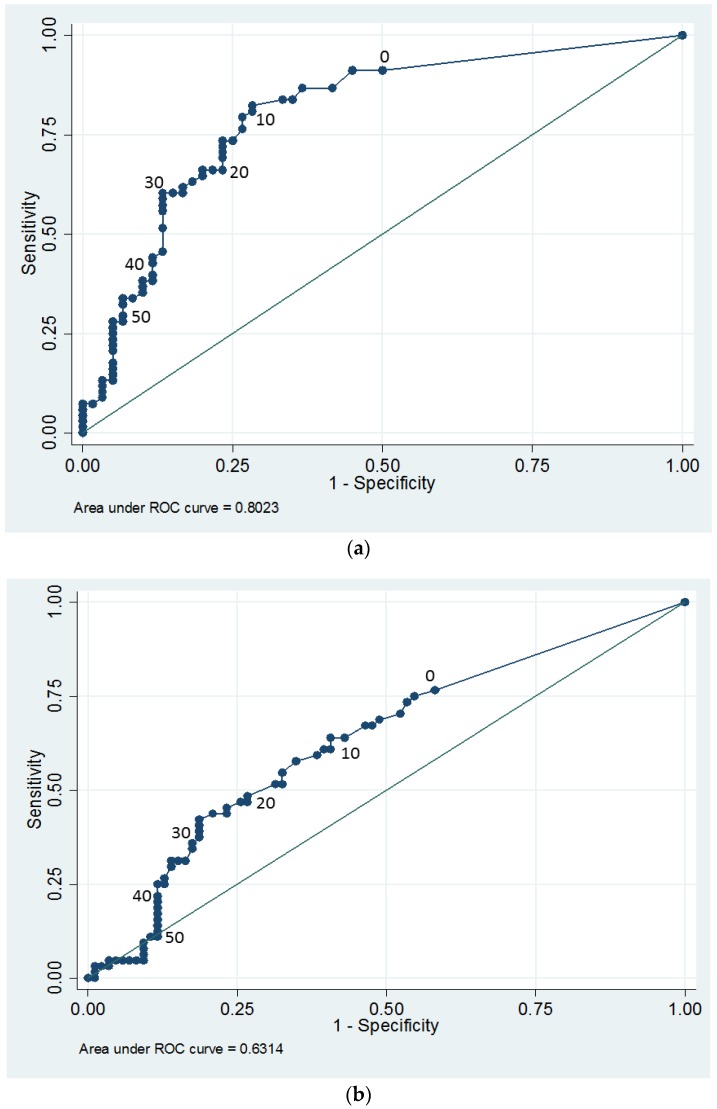
Receiver Operator Characteristics (ROC) Curve for relationship between extent of scarring and dog aggression (**a**) among males; (**b**) among females. A threshold of 10 or more scars was chosen for further analysis, for logistical and statistical reasons (see text). Numbers represent total number of scars in the 3 body zones: front legs, dorsum of head and lateral face, and muzzle and oral mucosa.

Based on the ROC curves (See Figure C1 in Appendix C), summed scar data from the 3 body zones were more reliable as a predictor of dog aggression among males (AUC = 0.82, 95% CI: 0.74–0.90) than females (AUC = 0.63, 95% CI: 0.54–0.73). We reviewed the ROC curves to determine if there was a single threshold of scarring in the 3 body zones that could be useful as an indicator of high risk of dog aggression. Several thresholds between 10 and 20 scars provided similar overall accuracy for classifying dogs as high risk. We chose to further evaluate the threshold of 10 scars for two reasons: logistically, it is easier to count fewer scars accurately, and statistically, a lower threshold reduces the likelihood of a false negative determination (i.e., truly dog-aggressive dogs are less likely to be misclassified as low risk in the screening process).

Table 5 contains the sensitivity, specificity, PPV, and NPV when using the threshold of 10 scars as a screening threshold for dog aggression. The PPV was 82% among males and 60% among females. This means that, among dogs with 10 or more scars in the three body zones, 82% of males and 60% of females could be expected to show dog aggression. The NPV was 74% for males and 65% for females indicating that, among dogs with nine or fewer scars in the three selected body zones, 74% of males and 65% of females would not show dog aggression. Because NPV and PPV are sensitive to the frequency of dog aggression in the population being analyzed, these NPV and PPV results should be generalized only to populations of dogs expected to be at similarly high risk for dog aggression. Therefore, these findings should only be applied to pit bull-type dogs seized from organized dog fighting investigations, and should not be applied to the general population of shelter dogs or to the general population of pit bull-type dogs.

## 4. Discussion

Among dogs seized in organized dogfighting investigations, a higher percentage of dogs with 10 or more scars on the front legs, dorsal and lateral head, and muzzle and oral mucosa were aggressive to dogs in their behavior evaluation than dogs with fewer than 10 or no scars. This finding suggests that extent of scarring on the three body zones is a fairly accurate tool that may be used in identification of dog aggressive dogs among populations seized in organized dogfighting investigations. However, the accuracy of this tool is imperfect. Some heavily scarred dogs were not dog aggressive and some unscarred or lightly scarred dogs were. Therefore, while 10 or more scars in the three body zones, especially on male dogs, could be useful in triage situations to quickly estimate risk of dog aggression, we recommend also gathering behavioral information about each dog before making disposition decisions.

The extent of scarring in this study ranged widely, from no scars to over 250. In our experience, this is representative of populations seized from dogfighting investigations. The majority of scarring was concentrated on the front legs, dorsal and lateral head, and muzzle and oral mucosa, consistent with findings that these zones are associated with organized dogfighting as opposed to spontaneous, non-organized fights between dogs [4].

Aggressiveness to dogs also varied widely, from non-aggressive, friendly behavior to highly directed, intense aggression. Overall in this population, which did not include puppies, 53% of dogs displayed either classic dogfighting behavior to the model dog or aggression to the real dog. Again, in our experience this is representative of the range of behavior found in dogs from organized dogfighting populations, based on results of the standardized behavior evaluation described herein.

There was a clear association between extent of scarring and aggression to dogs, with a steady increase in proportion that were aggressive as scar count increased. However, it is critical to note that 28% of dogs with no scars were aggressive to dogs, whereas 22% of dogs bearing 40 or more scars were not. Therefore, while scar count may be an aid in identifying dog aggressive and non-dog aggressive dogs from fighting cases, it is prone to both false negatives and false positives. Therefore, a scar count should not be utilized in isolation when making disposition decisions but in combination with observation of behavior towards dogs including handler and kennel staff reports and careful interactions with other dogs, as in the standardized behavior assessment described above.

The lowest proportion of dog-aggressive dogs was found among unscarred males, followed by unscarred females. These dogs may have been used for breeding purposes and therefore not fought extensively, if at all. Alternatively, they may have been dogs who were not yet rolled in a training fight, or they may have been kept by the owners as status or pet dogs and not for fighting. Many heavily scarred dogs of both sexes were also not aggressive to other dogs, however. These dogs may have been used to train other dogs or they might have been poor fighters but kept for breeding purposes due to their bloodline or fecundity. Conversely, many unscarred or lightly scarred dogs were dog aggressive. Some of these dogs may have been particularly good fighters who were able to disable their opponents quickly while sustaining few injuries. Others may not have been fought much yet, if at all. Still others may have been kept for breeding, or as pet or status dogs and not fought despite their dog aggression.

While it is possible that some older injuries had healed sufficiently such that they may be difficult to visualize upon examination, the severity of wounds associated with dogfighting likely result in scarring that remains visible over time, either as an area of alopecia or depigmented hair coat (white hair regrowth). All visible indications of scars and wounds were counted in this study, thereby utilizing a method available to any veterinarian performing such an examination.

We also must consider that any behavior evaluation protocol is not perfectly accurate at identifying dog aggressive dogs. It is likely that some dogs who were truly dog aggressive did not appear so in their behavior evaluation. Potential reasons for false negatives include dogs fearing the evaluation situation or the evaluators, masking their “normal” behavior. We often see very fearful dogs in fighting dog populations, likely because they spend much of their lives chained in a yard with little experience of the outside world. In addition, the evaluation context may have lacked the necessary stimuli to trigger aggressive behavior in some dogs (e.g., an active, aggressive opponent; a fighting pit). Using a model dog may have limited responses to aroused or precursory dogfighting behavior because the model does not give the dog appropriate feedback that may lead to display of classic dogfighting behavior. Similarly, the stimulus dog test was a controlled situation conducted on leash for just a few minutes with a stimulus dog that was generally rather tolerant and not dog-aggressive. There may have been more aggressive responses from the test dogs had we let the interaction continue longer, or not controlled the dogs on leash, or had the stimulus dog been more dog-reactive. Some fighting dogs may need such feedback from the other dog to initiate aggression, while others do not.

Relatively few dogs were categorized as aroused in this study, and of those, no clear association with scarring was evident (see Table S1). Aroused was a rather difficult behavior to categorize. Assertive, aroused mounting behavior, and sexually-motivated mounting behavior can be difficult to discern without allowing the dogs to potentially consummate a sexual act. We did observe some sexual mounting behavior directed towards the stimulus dogs even though the test dog and stimulus dog were always of the same sex. This is not unusual among dogs. For ethical reasons, we did not permit the male dogs to attempt sexual behavior toward the stimulus dogs in order to discern arousal from sexual mounting, which we categorized as not aggressive. Of dogs that displayed precursory dogfighting behavior to the model dog, as defined above, there was some association with amount of scarring, but not a clear trend (see Table S1). All dogs that displayed precursory fighting behavior to the model dog in this study were then introduced to a stimulus dog, and behavior with the stimulus dog took precedence in the analysis. We recommend cautious introductions to real dogs for more information when precursory fighting behavior is observed with a model dog.

In order to obtain a scar count, we recommend that veterinarians involved in dogfighting cases complete a canine skin wound and scar body diagram, otherwise known as a “scar chart”, as part of a complete forensic medical examination (see Figure S1). A scar count can then be obtained in the three body zones identified as being most consistent with organized dogfighting, based on the completed chart. Particular attention should be paid to whether there are 10 or more scars present in these three body zones. Ten scars was not only a statistically valid threshold in the data in this study, but is also a reasonable number for veterinarians to count accurately.

In a situation where risk of dog aggression must be estimated quickly, dogs with 10 or more scars in the three body zones, especially males, may be considered at high risk. However, because scar count appears to be a useful but imperfect predictor of dog aggression, we recommend gathering behavioral information in multiple contexts and with a variety of stimulus dogs to supplement scar count whenever possible, particularly before making disposition decisions. In addition to handler and kennel staff reports and a standardized behavior evaluation as described above, we recommend that dogs who do not display aggression or who display ambiguous behavior with the stimulus dog should be observed in controlled interactions with a variety of other dogs of both sexes. Interactions should include meeting on a leash and carefully refereed socialization sessions (on muzzle as conditions warrant) in order to ascertain whether a lack of aggression in the evaluation was an effect of the particular stimulus dog chosen, the effect of the evaluation context, or is a behavioral trait of the dog itself.

## 5. Conclusions

Among pit bull-type dogs seized in investigations of organized dogfighting, there was a significant but imperfect relationship between extent of scarring on the front legs, dorsal and lateral head, and muzzle and oral mucosa with dog aggression exhibited in a standardized behavior evaluation. Dogs with 10 or more scars in these body zones were more likely to display dog aggression than dogs with fewer or no scars. However, about one-quarter of unscarred dogs were dog aggressive and about one-fifth of dogs with 40 or more scars were not. Therefore, we recommend that behavioral information be collected in addition to scar count when making disposition decisions in order to make more accurate predictions and informed decisions. We must emphasize that our findings only apply to dogs with confirmed dogfighting origins, and should not be applied to dogs who do not originate from dogfighting cases.

## Figures and Tables

**Table 1:** Definitions of behavioral categories recorded during the model dog and stimulus dog tests in the behavior evaluation. If the test dog displayed classic dogfighting behavior to the model dog, that behavior was recorded and testing was ended. Otherwise, the test dog was introduced to the stimulus dog and behavior with the stimulus dog was recorded.

**Model Dog Test**	
**Behavioral Category**	**Observed Behaviors**
Not aggressive	Fearful, submissive, friendly, playful, neutral, or sexual behavior
Aroused	Displays highly focused, tense behavior towards the model dog May display confident, assertive posture (tail up, ears forward, may have hackles up) May jump up on model dog, place chin or paws on model’s back (a “chin over” or a “stand over”) or mount the model, but does not show teeth, growl or try to bite
Classic dogfighting behavior	Rushes in, immediately aggressive Knocks model dog down and lies over it, clasping with front legs (“lying over”) Bites, holds and shakes in model dog’s head/neck region Usually requires a breakstick or waiting until the dog regrips to remove the model dog
Precursory dogfighting behavior	Displays elements of classic fighting dog behavior, especially lying over, but not the full behavioral suite The dog may not rush in and immediately aggress and/or the dog may not bite, hold and shake the model dog’s head/neck region May lie over, clasp, growl with no bite or bites with little to no force May lie over and growl then mount May take time to escalate to aggression, may display investigation or assertive posturing behavior first May lie over and bite in various locations over the model dog’s body (not focused on head/neck)
Other aggression	Displays aggression that is fear-related, or is a socially appropriate and short-lived correction in response to the model dog being pushed at the test dog or being positioned over the test dog in an assertive manner
**Stimulus Dog Test**	
**Behavioral Category**	**Observed Behaviors**
Not aggressive	Fearful, submissive, friendly, neutral, or sexual behavior
Aroused	Displays highly focused, tense behavior towards the stimulus dog. May display confident, assertive posture (tail up, ears forward, may have hackles up). May jump up on stimulus dog, place chin or paws on its back (a “chin over” or a “stand over”) or mount it, but does not show teeth, growl or try to bite.
Offensive aggression *	Growls, lunges, snarls, snaps, bites or tries to bite, none of which appear to be fear-related.
Other aggression	Displays aggression that is fear-related, or is a socially appropriate and short-lived correction in response to the stimulus dog’s assertive or unruly behavior

* It was not ethical to allow an aggressive test dog unrestricted interaction with the stimulus dog, therefore any offensive aggression displayed by the test dog was sufficient to halt further testing and was categorized as “Offensive Aggression”.

**Table 2:** Characteristics of study dogs eligible for analysis (N = 252).

Characteristics	N	%
Sex		
Female	136	54.0
Male	116	46.0
Age		
6 months to <3 years	100	39.7
≥3 years	152	60.3

**Table 3:** Scar count by body zone. Data indicate the percentage of dogs having the indicated scar count in each zone (N = 252).

	Scar Count *			
Zone	None	1–9	10–39	40+
Dorsal and lateral head	43	26	29	2
Eye and periorbital region	80	20	0	0
Pinnae	67	31	2	0
Muzzle and oral mucosa	49	27	21	3
Dorsal and lateral neck	84	15	<1	0
Ventral neck and chest	68	29	3	0
Scapular region	82	18	<1	0
Front legs	37	24	34	6
Thoracic and lumbar spine	96	4	0	0
Lateral thorax and abdomen	92	8	0	0
Ventral thorax and abdomen	90	10	0	0
Hind legs	54	36	10	0
Pelvis and tail	80	20	0	0
Total from dorsal and lateral head, the muzzle and oral mucosa, and front legs	31	16	31	23
Entire body	29	14	23	34

* Includes recent wounds.

**Table 4:** Dog-directed arousal and aggressive behavior by scar count in the three body zones most commonly injured in organized dogfighting. Levels of scarring are significantly different from each other if the confidence intervals do not overlap. Fisher’s Exact *p* for overall association between scar count and behavior was <0.001 for males and 0.05 for females.

Behavioral Response ** (% of Row, 95% CI)				
Total Scar Count *	N	Not Aggressive or Aroused	Aroused	Aggressive
All Dogs				
Total sample	252	39 (33–45)	8 (5–12)	53 (46–59)
No scars	77	61 (49–72)	10 (5–19)	29 (19–40)
1–9	40	48 (32–64)	15 (6–30)	38 (23–54)
10–39	77	29 (18–40)	5 (1–13)	66 (55–77)
≥40	58	17 (9–29)	5 (1–14)	78 (65–87)
Male dogs only				
No scars	31	71 (52–86)	10 (2–26)	19 (7–37)
1–9	18	28 (10–53)	33 (13–59)	39 (17–64)
10–39	34	24 (11–41)	0 (0–10) ^†^	76 (59–89)
≥40	33	6 (1–20)	6 (1–20)	88 (72–97)
Female dogs only				
No scars	46	54 (39–69)	11 (4–24)	35 (21–50)
1–9	22	64 (41–83)	0 (0–15) ^†^	36 (17–59)
10–39	43	33 (19–49)	9 (3–22)	58 (42–73)
≥40	25	32 (15–54)	4 (0–20)	64 (43–82)

* Total number of scars (including wounds) in the three body zones: the front legs, dorsal and lateral head, and muzzle and oral mucosa; ** Response based on combined results from testing with model dog and stimulus dog. Dogs categorized as Aroused did not display Classic Dogfighting Behavior with the model dog and were Aroused when they met the stimulus dog. Dogs categorized as Aggressive displayed Classic Dogfighting Behavior to the model dog and/or Aggressive behavior to the stimulus dog. See text and Table 1 for complete behavioral definitions; ^†^ One sided 97.5% confidence interval.

**Table 5:** Reliability of using 10 or more scars (in the three body zones most commonly injured in organized dogfighting) as a screening criterion for inter-dog aggression. Sensitivity, Specificity, Positive Predictive Value (PPV), and Negative Predictive Value (NPV) are provided by sex of dog, with 95% confidence intervals.

	Male Dogs	Female Dogs
Sensitivity	81% (70%–89%)	63% (50%–75%)
Specificity	75% (60%–86%)	62% (50%–73%)
PPV	82% (71%–90%)	60% (48%–72%)
NPV	74% (59%–85%)	65% (52%–76%)

## Supplementary Materials

The following are available online at www.mdpi.com/2076-2615/6/11/72/s1, Figure S1: Existing scars and injuries chart template. Table S1: Behavioral response to model dog by scar count. Total scar count represents the total number of scars in the 3 body zones: dorsum of head and lateral face, muzzle and oral mucosa, front legs. Behavioral response data are given as percent of the row. Table S2: Dataset used for statistical analysis in this study.
